# From empirical imprinting to programmable synthetic receptors: a perspective on data science and machine learning for molecularly imprinted polymers

**DOI:** 10.1039/d6py00493h

**Published:** 2026-08-26

**Authors:** Ahmad Alsholi, Shreya Tiwari, Jiyizhe Zhang, Marloes Peeters

**Affiliations:** a Department of Chemical Engineering, University of Manchester Nancy Rothwell Building Manchester UK marloes.peeters@manchester.ac.uk

## Abstract

Molecularly imprinted polymers (MIPs) are porous materials generated by templated polymerisation, in which functional and crosslinking monomers are organised around a target molecule and fixed within a polymer network to create high-affinity, selective recognition sites for the chosen analyte. The appeal of MIPs lies in their robustness, low-cost and chemically versatile nature, in addition to directly encoding selective molecular recognition into polymer networks while retaining the thermal, mechanical, and processing advantages of synthetic materials. Yet, despite being discovered a century ago, MIP development remains dominated by empirical, target-by-target optimisation. The field therefore presents a compelling challenge for data science and machine learning: MIP performance emerges from a high-dimensional coupling of material, polymer synthesis and processing parameters but this complex design space is still only sparsely sampled. In this Perspective, we argue that the next step for MIPs is not simply better prediction of pre-polymerisation interactions, which is traditionally used to predict optimal composition. Pre-polymerisation metrics are weak predictors of functional performance once MIPs are synthesized and applied; therefore, a broader shift towards data-driven polymer design which considers manufacturing constraints from the outset is needed. We discuss our future vision for the field and how structured datasets, high-throughput screening, computational modelling, Bayesian optimisation and interpretable machine learning enable the move from empirical recipes towards programmable synthetic receptors. Sensing and sustainable manufacturing routes will be covered, placing particular emphasis routes on exploiting the robustness of MIPs to facilitate high-throughput production and biocompatibility challenges.

## Abstract 2

Molecularly imprinted polymers (MIPs) are porous materials generated by templated polymerisation, in which functional and crosslinking monomers are organised around a target molecule to create high-affinity, selective recognition sites.

## Graphical abstract

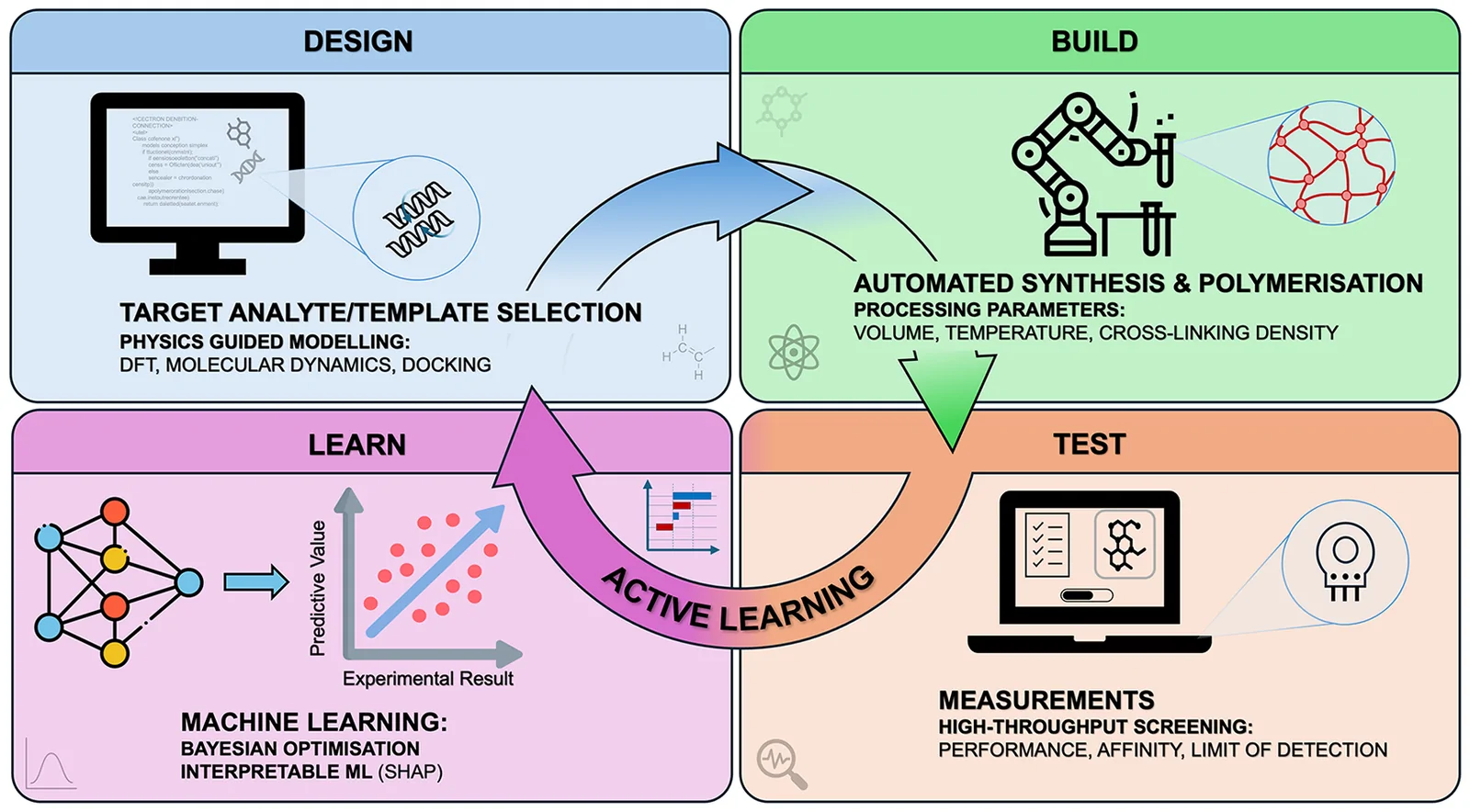

## 1. Introduction

**Fig. 1:**
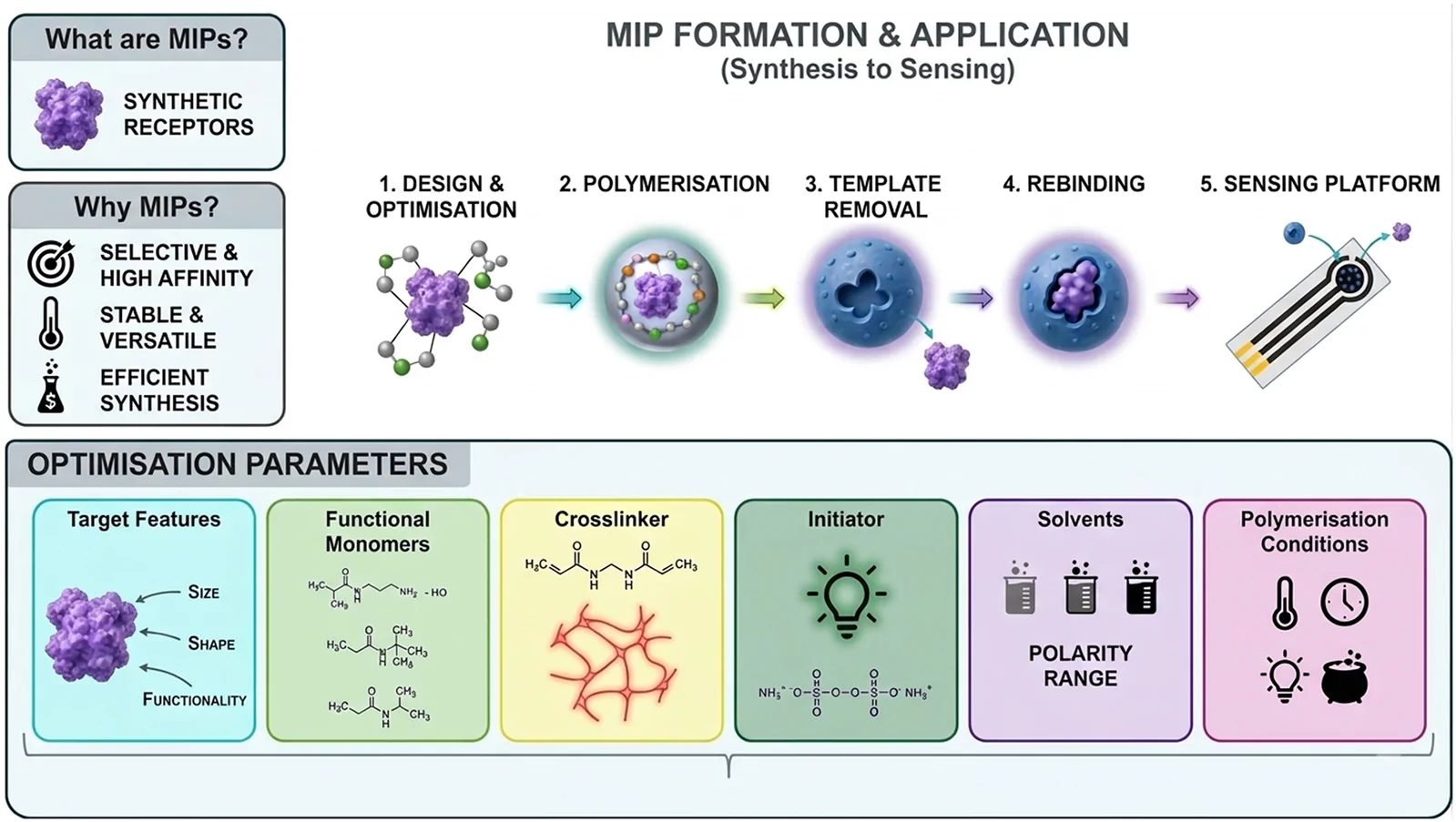
Molecular imprinting process and key factors governing MIP performance. Functional monomers assemble around a template, are polymerised and crosslinked, and the template is removed to create selective binding sites. Target properties, monomers, crosslinker, solvent and polymerisation conditions collectively determine recognition-site structure, accessibility and final performance.

Molecularly imprinted polymers (MIPs) are porous materials generated by templated polymerisation, in which functional and crosslinking monomers are organised around a (dummy) template and locked within a polymer network to create high-affinity, selective recognition sites for the chosen analyte.^1^ Once optimised, these materials can rival the specificity and selectivity of natural receptors (*e.g.* antibodies, enzymes) while offering significant advantages in terms of versatility, robustness, and cost-effectiveness.^2^ Therefore, MIPs have previously been coined as “plastic antibodies” or “antibody-mimics” and efforts have concentrated on replacing natural receptors with MIPs in existing applications. However, MIPs can exhibit multiple functionalities that go beyond natural recognition, facilitating novel applications in drug delivery and theragnostic applications where they might find a niche market.^3^ The molecular imprinting process and the principal formulation and synthesis variables that determine recognition-site structure and accessibility are summarised in Fig. 1.

Significant barriers to the commercialisation and translation of MIPs remain, particularly the lack of robust, reproducible and scalable processes for integrating them into functional platforms, including sensors and microfluidic devices.^4^ The next generation of MIP-based applications is moving beyond single-analyte proof-of-concept demonstrations to multimarker, multifunctionality and manufacturable systems. The central challenge is then no longer to simply bind a target, but how to develop a high affinity polymer whose recognition behaviour is not influenced by processing conditions and remains reproducible under realistic operating conditions. This ability would provide exciting opportunities in terms of cost-effective high-throughput production *via* printing methods, which are currently underexplored for MIP manufacturing.

Previous reviews have surveyed computational approaches for rational MIP design, including quantum-chemical calculations, molecular docking, molecular dynamics and, increasingly, machine-learning methods. These studies have provided important insights into template and functional-monomer selection and have highlighted the limitations of relying on pre-polymerisation models alone. However, their emphasis has generally been on the early design stage of MIP development. Less attention has been given to how computational and data-driven methods can connect molecular design with polymerisation, processing, device integration and application-level performance.^5,6^

Here, we adopt a broader and deliberately forward-looking perspective. Rather than listing individual algorithms or published AI-assisted MIP studies, we consider how the entire development pipeline can be reorganised as an integrated design–build–test–learn cycle linking molecular design, synthesis, processing, application-relevant testing and iterative experimental learning. Data science and artificial intelligence are particularly well suited to this challenge because MIP development involves multivariate design spaces that are difficult to interrogate empirically but can become amenable to structured learning when standardised and sufficiently complete datasets are captured. We therefore focus on three interconnected opportunities: establishing standards for MIP data and reporting; applying AI to polymer design, including inverse design; and using data-driven methods to guide synthesis, processing and experimental optimisation. We focus particularly on sensing applications, where the limitations of current trial-and-error workflows are most apparent, while also considering biocompatibility as an additional design constraint for MIPs intended for *in vivo* use.

## 2. Current bottlenecks of MIP design

Despite the steady growth of their applications, most MIP development follows a familiar route: choose one target, select promising monomers based on previous literature and/or computationally-guided design, followed by synthesizing a small number of candidates with varying composition and identifying the best performer experimentally.^7^ With literature expanding and resources such as MIPDatabase (https://mipdatabase.com) available to help guide design, this approach remains time-consuming and resource-intensive, which is not compatible with sustainable synthesis strategies.^8–10^

While inverse design approaches have transformed material design, there are two main reasons why trial-and-error design of MIPs persists. First, affinity and selectivity are not only governed by the interactions between template and functional monomer(s) but by a complex design space which includes polymerization parameters, solvent effects, particle growth, network rigidity, template removal, and the format in which the material is used. As a result, strategies that overprioritise the pre-polymerisation complex often fail to predict functional performance in the final material. What appears to be promising compositions in these simplified models may be lost during polymerisation, changed by network heterogeneity, or binding sites can become inaccessible after processing and device integration. This issue is particularly pressing for their application; while embedding MIPs in for instance solid-phase cartridges in fairly straightforward, this is not the case when integrating materials into sensors.^11^ We typically evaluate performance of the MIP materials with batch rebinding experiments or analytical techniques (*e.g.* surface plasmon resonance, isothermal titration calorimetry) that measure under controlled conditions. However, MIPs in a sensor are influenced by many factors that impact on apparent performance such as immobilisation chemistry, swelling, layer thickness, fouling, and transduction mode. Therefore, the “optimal” MIP in a screening assay is not necessarily the best MIP once embedded into an electrode coating or integrated microfluidic device.

Second, there is a lack of sustainable high throughput discovery of high-performance materials and manufacturing workflows. This contrasts with other synthetic receptors such as aptamers, where iterative selection platforms (*e.g.* SELEX), combined with automation, microfluidic handling and high-throughput sequencing, have enabled the parallel integration of very large candidate libraries for direct optimization of affinity and specificity.^12^ For MIPs, several routes towards parallelised or more scalable production have been reported, including combinatorial library screening in microtiter-plate formats and droplet-based microfluidic approaches for controlling particle size and formulation.^13,14^ These advances show that higher-throughput MIP discovery is feasible, but they remain specialized and have cumbersome purification procedures, limiting their adoption across the field.

More importantly, these methods do not fully resolve the linked challenges of yield, scalability, reproducibility and transfer to final application formats. Solid-phase synthesis, for example, can improve affinity-based purification and particle uniformity, but often at the cost of low absolute yield and cumbersome separation workflows.^15^ Automated systems demonstrate proof of principle for reproducible production, yet they have not become routine community tools.^16^ More generally, increasing throughput alone is not enough if the resulting materials cannot be manufactured efficiently, integrated into devices, or produced with acceptable economic and environmental costs. These issues make clear that manufacturing should not be treated as a downstream translational step, but as part of the MIP design space itself. Processing history, including solvent exposure, shear, thermal stress, purification, filler dispersion and polymer architecture, can all influence whether recognition sites remain accessible and functional in the final material. This is particularly important for applications that require integration into coatings, composites, membranes, cartridges, microfluidic devices or printable formulations, where the material must satisfy multiple performance constraints simultaneously. Although optimisation of MIP-based sensors often focuses on component selection, including the choice and ratio of functional monomers, crosslinker monomers and template, synthesis and processing parameters play an equally important role and should be captured in datasets intended for machine learning (ML). The central challenge is therefore to understand how polymer chemistry, synthesis, processing, device integration and biological exposure combine to determine function in the final application. Apparent affinity measured under idealised batch-rebinding conditions may not be retained following immobilisation, processing or incorporation into a sensor or other device. Translational constraints should therefore be encoded explicitly within the design space, rather than evaluated only after affinity optimisation.

This requires datasets and models that connect formulation-level inputs with batch-level process records, intermediate material properties and final application outcomes. Such datasets must capture variability to distinguish formulations that perform well under ideal laboratory conditions from those that can be manufactured reproducibly. For biological applications, the same record should incorporate relevant exposure conditions and safety-related outcomes, including cytotoxicity, inflammatory responses, the effects of degradation products, biodistribution and clearance, where appropriate. The objective is therefore to develop high-affinity polymers whose recognition behaviour is retained after processing and remains reproducible under realistic operating conditions, thereby enabling scalable and cost-effective manufacturing approaches.

**Fig. 2:**
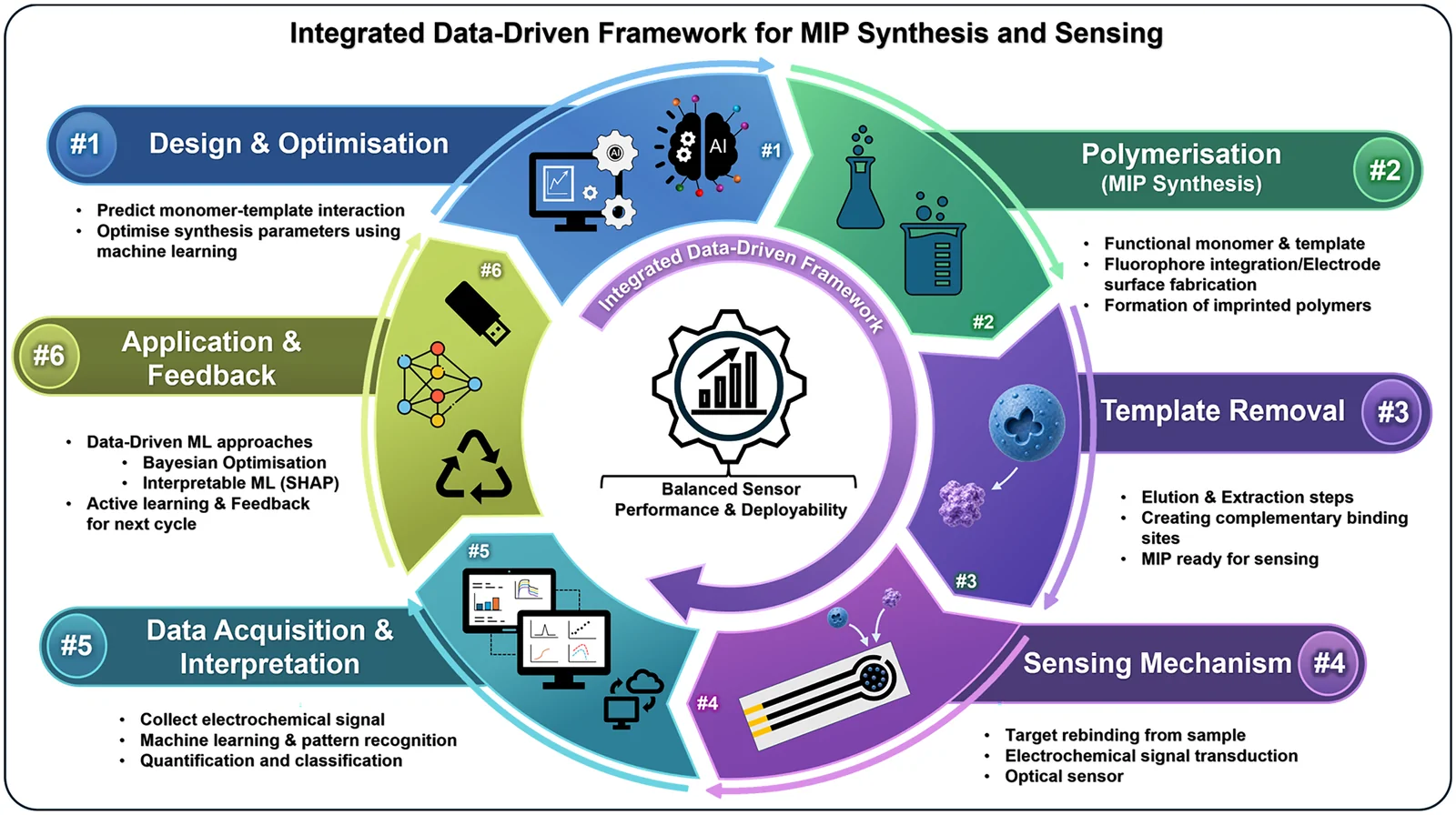
Integrated data-driven framework for molecularly imprinted polymer (MIP) development. The iterative workflow links computational design and optimisation, polymer synthesis, template removal, sensing, data acquisition and interpretation, and application feedback to improve sensor performance and guide subsequent design cycles.

These limitations point to the need for a different design framework: one that learns directly from experimentally measured performance under realistic conditions, while still retaining enough chemical interpretability to guide future synthesis. This shift can be organized by the design–build–test–learn (DBTL) cycle. In this framework, MIP development commences by defining the desired target receptor and optimal recipe, proceeds through synthesis and processing under controlled conditions, evaluates material and application-level using high through-put assays, and then uses the resulting data to update the next round of the design. This reframes molecular imprinting from a linear, target-specific optimization problem into an iterative polymer discovery workflow in which recognition, manufacturability, sustainability and end-use performance are optimised together. Overall, this will lead to identification of design rules that are transferable across targets and formats (Fig. 2).

The central challenge is no longer simply to identify monomer combinations that bind a target in principle, but to understand how polymer chemistry, synthesis, processing and manufacturing constraints combine to generate robust function in the final material. This is precisely where data science and machine learning could provide a step change, provided that future datasets are structured to capture not only successful formulations, but also failed experiments, process history and application-relevant performance.

## 3. A design–build–test–learn (DBTL) framework for data-driven MIP discovery

The DBTL framework provides a logical structure for data-driven MIP development by distinguishing four interdependent stages—design, build, test and learn—and connecting them within a closed, iterative cycle (see Fig. 2). In many current MIP workflows, target selection, synthesis, performance testing and data interpretation are undertaken largely as sequential activities, with limited feedback between stages. DBTL addresses this limitation by ensuring that data generated during testing and learning directly inform subsequent design, synthesis and processing decisions. The framework therefore clarifies four linked questions: what function should the MIP achieve, how should it be synthesised and processed, how should its performance be measured under application-relevant conditions, and how should the resulting data guide the next experimental cycle? This structure also clarifies where different computational and experimental tools contribute.

For instance, first-principle modeling is most often used in the design stage, polymer chemistry and process control dominate build, functional (high-throughput) assays define test, and Bayesian optimisation and ML drive learn. Table 1 provides an overview of these stages; current MIP practice, what data is required, and what opportunities there are in the field.

**Table 1:** Design–build–test–learn (DBTL) framework for data-driven and translational MIP development

DBTL stage	Current MIP practice	Data needed	AI/ML role	Opportunities	Translational constraints/outputs
Design	Monomer, template selection	Polymer descriptors, target profile, matrix, format	Define objectives, constraints and operating windows; prioritise candidate formulations	First-principles modelling, generative molecular design, ML for property prediction	Processability, stability, biocompatibility, intended device format, scalability and sustainability defined as design constraints
Build	Small empirical synthesis sets	Formulation, polymerisation, processing history	Optimise formulation and polymerisation conditions; model sources of batch variability	Controlled/high-throughput synthesis using automated/robotic tools	Yield, batch-to-batch variability, particle/film properties, purification, processing compatibility, scale-up and manufacturing reproducibility
Test	Rebinding, imprinting factor	Material- and application-level performance linked to formulation, batch, processing and device metadata	Identify performance-driving parameters; model trade-offs and probability of meeting specifications	Standardised assays, online monitoring, data-rich characterisation	Device-level sensitivity, response time, fouling, stability, matrix effects, pre-/post-integration performance and, where relevant, cytotoxicity, degradation, biodistribution and clearance
Learn	Retrospective interpretation	Structured datasets including failures, variability and translational outcomes	Extract design rules; quantify uncertainty; perform constrained/multi-objective optimisation and guide the next cycle	ML/Bayesian optimisation	Optimise affinity alongside reproducibility, manufacturability, device performance, safety, cost and sustainability rather than as a single objective

Alongside open-source tools, a range of existing platforms is increasingly used in academic and industrial materials development. These include polymer-informatics and molecular-modelling environments for candidate screening, such as BIOVIA Materials Studio, the Schrödinger Materials Science Suite and Polymer Genome-type platforms; design-of-experiments software, including JMP, Design-Expert and MODDE, for systematic exploration of formulation and processing variables; and Bayesian-optimisation or closed-loop experimentation platforms, such as Citrine Platform, MaterialsZone and Atinary, which can support experimental planning and selection of subsequent experiments. These tools, while not routinely used yet, can contribute across multiple stages of the DBTL cycle, from molecular design and structured data capture to process optimisation and iterative learning. However, their uptake in molecular imprinting remains limited, and wider adoption may be constrained by cost, interoperability, transparency and restricted access to underlying models.

### 3.1 Design: selection of analyte, template and target properties

The design stage defines the function of the MIP prior to synthesis. Following the DBTL framework, design should commence with the target-receptor profile: the analyte (or family of compounds) that needs to be recognised, the required affinity and selectivity, the matrix for recognition, final material format, and constraints on the system including stability, biocompatibility, processability and sustainability. The latter is crucial since the best monomer–template interaction combination *in silico* may not translate into the best performing material after polymerisation, template polymerisation, and platform integration. However, MIP informatics is not a trivial extension of existing computational workflows. Crosslinked and heterogeneous polymer synthesis remain difficult to represent and model, especially when local chemistry and morphology jointly determine function.

Quantum chemical calculations, molecular docking, and molecular dynamics are increasingly used to guide early design choices for MIP development. Density functional theory (DFT) is especially useful for early-stage candidate down-selection as it has been shown that calculated binding energies of the pre-polymerisation complex align with observed experimental ranking of formulations.^17,18^ Docking requires less-intense computational resources and is primarily used to identify binding sites between the template and functional monomers.^19^ Molecular dynamics (MD) extend this by simulating how these complexes evolve over time, making it possible to evaluate conformational flexibility and competitive solvent effects.^20^ A range of open-source computational tools can support these early design stages. For example, the quantum-chemistry package ORCA can be used for geometry optimisation, interaction-energy calculations and solvent modelling, providing molecular-level information that can support the selection of functional monomers and porogens.^21^ Molecular dynamics packages such as LAMMPS can extend these calculations by modelling pre-polymerisation mixtures, including molecular distributions, non-bonded interactions and hydrogen-bonding networks, thereby providing insight into the formation and stability of template–monomer assemblies.^22^ Open-source docking software such as AutoDock Vina offers a less computationally intensive means of predicting binding conformations and approximate binding affinities between templates and candidate monomers. Supporting packages, including AutoDock MGLTools and PyMOL, can facilitate molecular preparation and visualisation of predicted binding modes.^23^ Although workflow-management platforms such as AiiDA are increasingly used to automate high-throughput computational chemistry, their application to molecular imprinting remains limited. Table 2 provides a qualitative comparison of these tools mentioned, summarising their main function, accessibility, and inputs/outputs required. Open-source software can support most routine academic workflows, whereas integrated commercial platforms may be advantageous for more complex, automated or multiscale modelling tasks.

**Table 2:** Practical comparison of representative computational tools for data-driven MIP design

Tool	Main function	Learning curve	License	Inputs	Outputs
ORCA	Quantum chemical calculations (DFT)	Moderate	Open source (academic use)	Molecular structure, basis set, calculation settings	Optimised geometries, interaction energies
LAMMPS	MD simulations	High	Open source	Molecular system, force field, simulation parameters	Molecular trajectories, structural, and interaction analyses
AutoDock Vina	Molecular docking	Low-moderate	Open source	Target and ligand structures	Binding poses, estimated binding affinities
AiiDA	Workflow automation	Moderate-high	Open source	Computational workflows and software inputs	Automated workflows, reproducible datasets
Schrödinger	Molecular modelling and simulation	Moderate	Commercial	Molecular structures and simulation settings	Docking results, binding free-energy estimates, molecular simulation data
PyTorch/TensorFlow	Neural network development and training	Moderate-high	Open source	Training datasets, model architecture, and hyperparameters	Trained neural networks, predictions, learned representations and performance metrics

Epitope imprinting typically is a popular approach for developing MIPs for macromolecules, considering that imprinting with the full macromolecule is often not cost-effective or has issues around stability, and can support the selection of accessible peptide templates to enhance binding. For smaller analytes, we might consider dummy templates because of economic, health and safety reasons, or when we do not specifically want to bind one template but a family of compounds (such as a family of antibiotics for environmental monitoring), where modelling can be used to find suitable templates.^24^ However, physics-guided screening alone is not enough. By design, these methods typically capture a simplified representation of recognition before polymerisation, whereas practical MIP performance emerges only after network formation, template removal, and, often, integration into a sensing architecture.

A promising strategy is therefore the direct integration of mechanistic or equation-based models with a data-driven model. These hybrid mechanistic/data-driven models are widely used in chemical-process and bioprocess modelling, but their development for MIPs is particularly challenging. MIP performance depends on processes spanning several scales, including template–monomer interactions, solvent effects, crosslinking, network heterogeneity, and application-level behaviour. These processes are difficult to capture within a single sufficiently accurate mechanistic model, particularly for heterogeneous crosslinked polymers. Tightly coupled hybrid models therefore remain underexplored in MIP research, although empirical models and ML provide a practical step towards this goal. Prospective datasets linking molecular descriptors, synthesis conditions, material structure and functional performance will be required to support their development.

### 3.2 Build: synthesis, polymerisation and processing as design variables

**Fig. 3:**
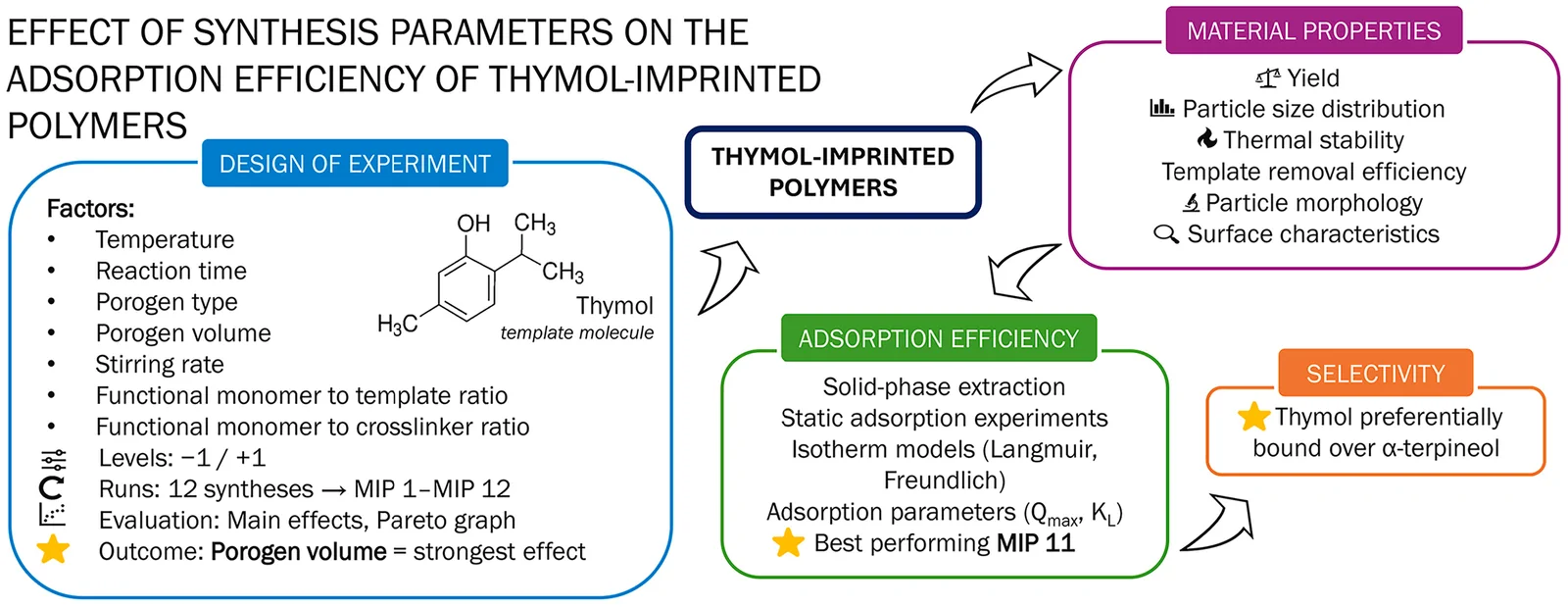
Example of thymol-imprinted polymer design space, showcasing the influence of synthesis parameters on MIP structure and binding performance. Synthesis conditions such as porogen volume, polymerisation time, temperature and agitation shape pore architecture, network rigidity and binding-site accessibility in molecularly imprinted polymers. This highlights that MIP performance, linked to the final network structure, is determined by polymerisation conditions and processing conditions. Reproduced from ref. 26 with permission from Elsevier, A. Šmídová, A. Křížová, E. Vyskočilová, M. Lhotka and L. Dolejšová Sekerová, *Polymer*, 2026, **354**, 129965, https://doi.org/10.1016/j.polymer.2026.129965, copyright 2026.

The build stage converts the proposed design into the polymer material, which is where many current workflows lose predictive control. Polymerisation generates the network topology, local crosslink density, binding site stability, pore architecture, and functional group orientation, factors that ultimately govern binding-site accessibility and mass transfer. This is particularly important for MIP synthesised by free radical polymerisation, where small differences in initiation rate, propagation kinetics, solvent environment and gelation can affect binding motifs.^25^ For instance, excess monomers used during the synthesis can generate heterogeneous, non-specific sites that lower the overall selectivity of the material.^26^ Similarly, porogen volume, polymerisation time, temperature and agitation can influence pore architecture, network rigidity, binding site integrity and mass transfer, as shown in Fig. 3.

This is underlined by experimental and factorial design, demonstrating that synthesis parameters, and in particular porogen volume, strongly influence pore structure and adsorption and thus govern binding site accessibility and quality.^26^ Moreover, template removal is a critical factor that is linked to binding site integrity and overall extraction efficiency.^27^ Downstream processing steps (*e.g.* washing, immobilization, and incorporation into platforms) can influence whether recognition sites remain accessible in a final format. This is especially relevant for applications in sensors, cartridges, or devices, where the material must retain recognition after being integrated into a functional architecture.

The build stage also determines whether MIPs can be manufactured reproducibly and sustainably. Sustainability further strengthens the case for rethinking how MIPs are designed and evaluated. Many current synthetic routes depend on significant solvent use, excess reagents, energy-intensive processing and inefficient template consumption, all of which become increasingly problematic as the field moves from laboratory-scale synthesis to scalable production. Tools such as AGREEMIP are therefore valuable because they provide a structured way to identify environmental hotspots in MIP production, including solvent demand, waste generation and energy use.^28,29^ However, this method is based on data extracted from a limited (50) selection of papers and provides a qualitative green chemistry approach, where the emphasis is on lab-scale synthesis and penalising the use of hazardous chemicals rather than considering mass balances. This framework could be expanded by including broader and more diverse indicators, such as energy intensity or energy efficiency. In addition, techno-economic analysis should be considered early rather than retrospectively if the aim is to develop scalable platforms with commercial potential.

### 3.3 Test: measuring functions under realistic conditions

The test stage determines whether the built materials meet the intended profile defined in the design stage. In traditional MIP development workflows this assessment often relies on batch rebinding experiments, imprinting factor, and apparent affinity or selectivity measured under controlled conditions. These measurements hold value but are not sufficient for data-driven design because they may not predict performance in the final application. A MIP optimised in a rebinding assay could behave differently when immobilized on an electrode, embedded in a hydrogel, incorporated into a membrane, or exposed to a complex matrix.

Testing should therefore capture both material-level and application-level performance. Measurements should be encoded using a hierarchical data structure that preserves the relationships among formulation, synthesis batch, processed material, device configuration and biological function. Multiple independently prepared samples may derive from the same batch and be evaluated under different operating or exposure conditions. Collapsing these results into a single mean value, such as affinity or limit of detection, would obscure sources of variability and the effects of processing that are critical to translation. Each outcome should therefore remain linked to the relevant formulation, batch, processing, device and testing metadata. The same structure should capture intermediate physicochemical and biological properties, including solubility, porosity, stability, degradation behaviour and, for biological applications, properties such as biocompatibility, biodistribution and clearance. These properties may be measured directly or predicted from molecular, formulation and material descriptors, enabling unsuitable candidates to be excluded earlier and subsequent experimental results to validate and refine the models.

These translational properties can then be represented within ML workflows as continuous objectives, categorical outcomes or feasibility constraints. Affinity and sensitivity, for example, may be treated as optimisation objectives, whereas cytotoxicity or inflammatory responses are defined by safety thresholds. Reproducibility can be represented using variance components, coefficients of variation or the probability of meeting a predefined specification across independently manufactured batches. Manufacturability may be described using yield, cycle time, sustainability impact, cost, and compatibility with scalable deposition or printing methods. Device integration should be assessed using paired measurements before and after immobilisation or fabrication, allowing models to learn how processing affects accessible binding sites, mass transport and signal transduction. Recording these outcomes separately would enable constrained multi-objective optimisation that balances affinity against other process factors.

The test stage is where the field needs stronger infrastructure aligned with the FAIR (findable, accessible, interoperable, and reusable) principles to explore data analytics and AI methods.^30^ MIP datasets are often small, but a dataset designed for learning should include the full experimental design space including poor-performing and failed formulations. Negative results are particularly valuable because they define the boundaries of the viable design process and help prevent ML models from overestimating performance. At present, much of the MIP literature is difficult to mine computationally because key information is inconsistently reported, including monomer and crosslinker ratios, solvent and porogen composition, polymerisation conditions, template-removal procedures, washing protocols, processing history and assay conditions. Without these metadata, it is difficult to determine whether differences in performance arise from receptor chemistry, network formation, post-processing, or measurement conditions.

High-throughput experimentation could transform this stage if coupled to standardised and application-relevant readouts. However, high-throughput binding assays should ideally be complemented by measurements of kinetics, non-specific binding, matrix effects and reproducibility; for sensors, screening should include device-level outputs rather than only solution-phase affinity. This would allow test-stage data to feed directly into the learn stage, where ML models and Bayesian optimisation can identify which material, synthesis and processing variables should be changed in the next DBTL cycle. This would also provide an opportunity for large language models which can extract historical data from literature to reveal broad trends and provide generalisable design rules. A priority for the field should therefore be to make new MIP datasets more structured, complete and machine-readable from the outset.

### 3.4 Learn: informed by Bayesian optimization and ML approaches

The learn stage uses the experimental results to update the next design and build decisions; this is where ML becomes critical. The DBTL framework uses ML to connect descriptors across scales: molecular structure, formulation, synthesis conditions, processing history, material properties, and application-level performance. Surrogate models are particularly valuable when experimental datasets are limited and generating new observations is costly. For example, Dykstra *et al.* trained a Gaussian-process model using a dataset of 72 electropolymerized MIP synthesis conditions for cortisol detection. The model was combined with global and local sensitivity analyses to quantify the influence of synthesis parameters, identify improved conditions and guide experimental validation, resulting in a reported 1.5-fold improvement in sensitivity.^31^ Bayesian optimisation is a natural extension of this approach and can encompass multi-objective formulations, enabling simultaneous optimisation of binding, selectivity, robustness, manufacturability cost, and sustainability. Bayesian optimisation is well-suited to MIP discovery because of the time-consuming and multidimensional nature of these experiments. By balancing exploration of uncertain regions with exploitation of promising formulations, Bayesian optimisation can prioritise the next most informative experiments while reducing the number of materials that need to be manufactured.

Importantly, interpretable ML tools such as Shapley Additive Explanations (SHAP) or related feature-attribution methods can then be used to identify which variables are truly driving performance. SHAP has been employed to interpret machine learning models, including decision trees, random forests and XGBoost, by identifying the relative importance of experimental variables, which improves the understanding of their impact on model predictions and guides feature engineering to improve predictive performance.^32^ In another study, SHAP has been combined with a physics-informed random forest model to analyse which engineered features were driving model predictions, and it demonstrated that the model reflected the underlying electrochemical behaviour rather than spurious statistical correlations.^33^ MIP datasets are often relatively small, tree-based models like decision trees, random forests and XGBoost combined with SHAP analysis, may provide a good balance between prediction accuracy and model interpretability. In practice, the balance between predictive accuracy and interpretability should depend on how the model output will be used within the DBTL cycle.

Interpretability is particularly important when model predictions are used to generate chemical design rules, select monomers or solvents, modify polymerisation conditions, or alter processing, manufacturing or biocompatibility strategies, because users must be able to assess whether the identified relationships are chemically plausible and transferable. This applies especially to the design stage, but also to the learn stage whenever model outputs directly determine subsequent design or build decisions. More complex, less interpretable models may be acceptable for lower-consequence tasks, such as preliminary candidate ranking, automated screening or prioritisation of experiments, provided that their predictive performance is robustly validated and that the consequences of an incorrect prediction are limited. The aim is therefore not to exclude black-box models, but to use more interpretable approaches when model predictions have important scientific or practical consequences, while allowing less interpretable models for lower-risk exploratory tasks.

This distinction matters because the learn stage has two complementary functions. First, it involves learning a predictive model: experimental data are collected and used to train an ML model that connects molecular, formulation, synthesis and processing descriptors with target material properties or application-level performance. Feature-attribution methods, sensitivity analysis and uncertainty-aware models can then identify which variables most strongly influence the response, such as monomer ratio, porogen volume, film thickness, extraction conditions, electrode modification or matrix effects. Second, the learned relationships can be used in the inverse direction: rather than predicting the performance of an existing formulation, the model proposes new polymers, formulations or experimental conditions expected to achieve specified target properties. The goal in MIP development is not only to predict better candidates, but to extract transferable design rules which can be applied to any type of analyte or application.^34^ In this way, the learn stage closes the DBTL loop by converting experimental observations into predictions, hypotheses and new experimental designs that inform the subsequent design and build stages.

## 4. DBTL in practice: MIP-based sensors as a test case

We focus here on sensing because it is one of the most active application areas for MIPs and clearly illustrates why design must be treated as a multiscale problem. In the design stage, the profile must include the transduction mode, matrix, target sensitivity, and operational constraints. In the build stage, polymerisation, immobilisation, electrode modification, film thickness and post-processing determine whether recognition sites remain accessible at the interface. In the test stage, performance must be measured using device-level outputs such as sensitivity, response time, regeneration, fouling resistance, and matrix tolerance, rather than affinity alone. In the learn stage, these coupled variables can be optimised using Bayesian optimisation and interpretable ML to connect material design with sensor-level performance.

For electrochemical sensing, as an example, performance depends not only on the MIP but also on conductivity, the presence of additives such as graphene or carbon nanotubes, their dispersion within a conductive matrix, and the efficiency of interfacial electron transfer.^33^ For optical sensing, performance depends on how binding is coupled to signal generation, for example through fluorescent probes, photonic structures or other optically active components embedded within the MIP matrix. In such systems, signal output can be strongly influenced by probe positioning, the local polymer and sample environment (*e.g.* pH, ionic composition) as well as quenching effects.^35^ Fabrication and deposition methods add another level of complexity as they determine binding at the sensor interface. For instance, it was shown that direct UV polymerisation of MIPs on electrodes achieved lower detection limits than drop-cast microparticle coatings, while also improving reproducibility and fabrication efficiency.^36^ Importantly, conventional metrics such as binding affinity or imprinting factor do not always translate into sensor-level performance indicators such as sensitivity, response time or limit of detection.^37^

A core limitation in current MIP sensor development is therefore the gap between material optimisation and device-level performance. These complexities highlight that optimisation of MIP-based sensors depends on multiple factors that cannot be captured by single-variable analyses and instead requires more systematic and data-driven approaches. In this context, approaches such as Bayesian optimisation can offer a practical way to explore these coupled variables more efficiently and better connect material design with device-level performance. It should also be clarified what “robustness” should mean for MIP-based sensors. MIPs are often described as robust alternatives to biological receptors because they generally tolerate harsher thermal and chemical conditions, can be stored at room temperature, have virtually “infinite” shelf life and are often assumed to show low batch-to-batch variation.^38^ Yet, reproducibility and manufacturability remain persistent barriers to translation. Recent literature emphasises that binding-site heterogeneity, the statistical nature of free-radical polymerisation, and poor control over film thickness or particle structure can all lead to inconsistent performance, particularly once MIPs are integrated into sensing platforms. In theory, electropolymerisation offers better control over film growth and direct integration onto electrode surfaces; however, reproducibility still strongly depends on polymerisation conditions, template extraction and film stability, and is therefore not necessarily better than multi-step functionalisation procedures. Moreover, this method is not directly compatible with high-throughput manufacturing, thus limiting commercial potential of such sensors.^39,40^ Variability in batch synthesis and limited process control further complicate large-scale production, highlighting the need for more standardised and scalable fabrication techniques.

These considerations become even more important for *in vivo* applications, where a high-performing MIP may still be unsuitable if its formulation, degradation products or residual monomers compromise biological compatibility. Biocompatibility is therefore better viewed as a design objective than as a late-stage filter. At present, however, biocompatibility remains much less understood than recognition performance, with evidence still dominated by short-term *in vitro* assays and a limited number of animal studies. Key questions around biodistribution, clearance, degradation and immunogenicity therefore remain unresolved, and future datasets should incorporate descriptors related to stability, surface charge and cytocompatibility if machine learning is to support truly multi-objective MIP design.^41^

## 5. Outlook and opportunities

Molecular imprinting provides a timely and compelling test case for data-driven polymer chemistry, a field with clear synthetic flexibility, strong application pull, and a design space that is too complex to optimise empirically. The next stage of progress is therefore unlikely to come from incremental refinement of individual recipes alone. Instead, the field needs to move towards transferable design knowledge by understanding how template chemistry, monomer selection, polymerisation, processing and application format combine to generate robust synthetic recognition. Embedding MIP development within a DBTL framework provides one practical route to this transition.

Using this framework, we have identified three key priority areas for the field. A first priority is the development of structured and standardised datasets and future datasets should capture successful and failed formulations, monomer and crosslinker ratios, template strategy, solvent and porogen composition, polymerisation conditions, template-removal protocols, processing history, material characterisation and application-level performance. Community reporting standards, curated databases and machine-readable metadata would therefore be as important as algorithm development itself.

A second priority is to connect modelling more directly to experimental performance. Although several studies now combine computational screening with experimental validation, and a smaller number use ML to predict MIP binding or performance from experimental data, genuinely hybrid design workflows are underexplored because robust representations of crosslinked polymers, network heterogeneity, and multiscale descriptors linking molecular interactions to material morphology and application-level performance are still lacking. This is particularly important if design rules are to become transferable across analytes, polymer formats and applications.

A third priority is to shift testing from simplified material-level assays towards application-relevant performance measurements. MIPs are often optimised using batch rebinding, imprinting factor or apparent affinity, but these metrics may not predict performance once the material is integrated into a sensor, membrane, cartridge, coating or biological system. Future DBTL workflows should therefore test MIPs in formats that reflect their intended use, capturing not only affinity and selectivity but also kinetics, matrix tolerance, fouling, stability, reproducibility, manufacturability and long-term function. This is essential if ML models are to optimise materials for real-world performance rather than for simplified surrogate readouts.

Many MIP researchers have limited experience with programming or ML approaches, and developing bespoke models may still require specialist expertise. However, open-source libraries and increasingly user-friendly software are lowering the barrier to entry. Wider adoption will therefore depend on accessible tools, appropriate training and interdisciplinary collaboration. Large language models and automated literature-mining tools may help extract historical information from published MIP studies, but they cannot overcome incomplete reporting, publication bias or the absence of negative results. The greater opportunity in using applying ML in MIP development is prospective: designing experiments that generate structured, machine-readable data suitable for learning based on increasing (automated) experimental throughput. High-throughput experimentation, automated synthesis, microfluidic screening and standardised assays could expand the accessible design space, provided that outputs are linked to material and application-level performance. Practical entry points could include electronic laboratory notebooks that help researchers structure experimental data, curated community repositories, example datasets and step-by-step Jupyter notebooks that allow users to test established workflows on small internal datasets. For example, Bayesian-optimisation packages designed for chemistry and chemical engineering may provide a more accessible starting point than lower-level libraries intended primarily for algorithm development. Developing new ML approaches suitable for MIP development is also demanded, such as multi-fidelity Bayesian optimisation, in which large numbers of rapid, lower-resolution measurements are combined with smaller numbers of higher-fidelity experiments. This could allow researchers to explore broader formulation and processing spaces while reserving resource-intensive experiments for the most informative candidates. However, its successful application will depend on clearly defining the relationship between fidelity levels and ensuring that lower-resolution measurements are sufficiently predictive of the final performance metrics.

Effective implementation of ML approaches and data science in MIP design will also require close collaboration: domain scientists should define the chemical question, design experiments, ensure data quality and assess whether model outputs are chemically plausible, while data scientists should support data structuring, model selection, validation, uncertainty analysis and reproducible implementation. Models and workflows should then be developed iteratively, with both groups jointly interpreting the results and deciding which experiments should follow.

The goal of data-driven MIP research is therefore not simply faster optimisation of individual formulations, but a shift from isolated target-specific demonstrations to a self-improving field in which each experiment contributes to shared design knowledge. If achieved, MIPs can move beyond “synthetic antibodies” towards programmable biomimetic receptors whose recognition, synthesis, processing and application performance are designed together.

## Author contributions

Ahmad Alsholi: writing – original draft, conceptualisation, formal analysis. Shreya Tiwari: writing – original draft, data curation, formal analysis. Jiyizhe Zhang: writing – review and editing, conceptualisation, supervision. Marloes Peeters: writing – original draft, conceptualisation, supervision, funding.

## Conflicts of interest

The authors have no conflicts of interest to report.

## Data Availability

No new data were generated or analysed in this study. All information discussed is based on previously published literature cited in the article.
